# Active learning driven prioritisation of compounds from on-demand libraries targeting the SARS-CoV-2 main protease†

**DOI:** 10.1039/d4dd00343h

**Published:** 2025-01-08

**Authors:** Ben Cree, Mateusz K. Bieniek, Siddique Amin, Akane Kawamura, Daniel J. Cole

**Affiliations:** a School of Natural and Environmental Sciences, Newcastle University Newcastle Upon Tyne NE1 7RU UK daniel.cole@ncl.ac.uk

## Abstract

FEgrow is an open-source software package for building congeneric series of compounds in protein binding pockets. For a given ligand core and receptor structure, it employs hybrid machine learning/molecular mechanics potential energy functions to optimise the bioactive conformers of supplied linkers and functional groups. Here, we introduce significant new functionality to automate, parallelise and accelerate the building and scoring of compound suggestions, such that it can be used for automated *de novo* design. We interface the workflow with active learning to improve the efficiency of searching the combinatorial space of possible linkers and functional groups, make use of interactions formed by crystallographic fragments in scoring compound designs, and introduce the option to seed the chemical space with molecules available from on-demand chemical libraries. As a test case, we target the main protease (Mpro) of SARS-CoV-2, identifying several small molecules with high similarity to molecules discovered by the COVID moonshot effort, using only structural information from a fragment screen in a fully automated fashion. Finally, we order and test 19 compound designs, of which three show weak activity in a fluorescence-based Mpro assay, but work is needed to further optimise the prioritisation of compounds for purchase. The FEgrow package and full tutorials demonstrating the active learning workflow are available at https://github.com/cole-group/FEgrow.

## Abstract 2

Software for building congeneric series of compounds in protein binding pockets is interfaced with active learning and searching of on-demand chemical libraries to automate the *de novo* design of compounds targeting the SARS-CoV-2 main protease.

## Graphical abstract

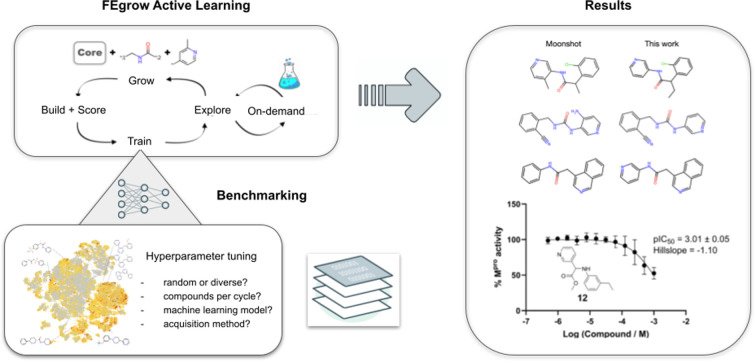

## Introduction

Recent advances in structural biology, from sample preparation, to synchrotron infrastructure and data analysis pipelines, have transformed the throughput of protein–ligand complexes available to inform drug discovery campaigns.^1^ When soaked with carefully designed compound libraries,^2^ the numbers of small molecule (or fragment) structural hits can reach tens or hundreds against a single therapeutic target.^3^ A frequently employed next step is to attempt to grow and/or link the hit compounds, using either custom synthesis^2^ or ordering from catalogues of purchasable compounds.^4,5^ However, chemical space is vast such that even choosing follow-up compounds for purchase from on-demand libraries, such as the readily accessible (REAL) Enamine database^6^ (>5.5 bn compounds in 2022), becomes highly non-trivial.^7^

As such, attention is turning to cheminformatics and machine learning based algorithms for structure-based *de novo* hit expansion, linking and merging.^8^ A wide range of approaches are available to build from initial structural biology data, including DeepFrag^9^ that identifies promising fragments for addition to an input bound ligand, using a deep convolutional neural network, and DEVELOP^10^ that combines 3D pharmacophoric constraints from the binding pocket with a graph-based deep generative model for R-group and linker design. The SILVR method enables an equivariant diffusion model to be conditioned to generate molecules based on a reference structure, such as a fragment from a crystallographic screen.^11^ The V-SYNTHES approach makes use of on-demand libraries for hit-finding by decomposing compounds from purchasable databases into reactive scaffolds and synthons, and using the highest scoring docked fragments as seeds for further growth.^12^ One particularly noteworthy example is the use of fragment merging to design hits against the nonstructural protein 3 (NSP3) of the severe acute respiratory syndrome-coronavirus-2 (SARS-CoV-2).^13^ Fragments from a crystallographic screen were merged using the Fragmenstein package,^14^ ensuring placement of molecular substructures onto the original fragments, and subsequently used as templates for searching on-demand chemical space. In this way, fragments were rapidly elaborated into a 0.4 μM hit (representing a >400-fold improvement in affinity).

**Fig. 1:**
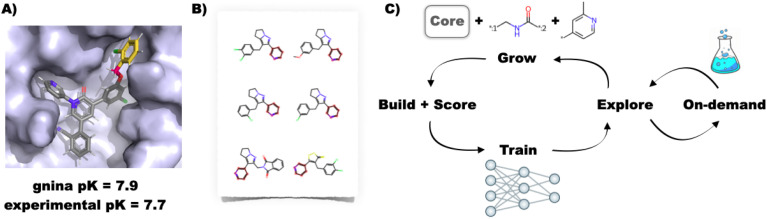
(A) Example building and scoring of a SARS-CoV-2 inhibitor^20^ using the interactive FEgrow workflow.^15^ The fixed core (grey) is extended using a user-defined, flexible linker (pink) and R-group (yellow), and scored using gnina.^19^ (B) Compound libraries with substructures that match the rigid core can now be automatically grown and scored, treating the rest of the molecule as fully flexible. (C) Proposed active learning cycle. Compounds are grown, built in the binding pocket and scored with FEgrow. The outputs are used to train a machine learning model, which is used to select the next batch of compounds. Optionally, the chemical space can be seeded using compounds available from on-demand chemical libraries.

While extremely promising, all of the above *de novo* design approaches suffer from some combination of the following issues: (i) reliance on an approximate classical molecular mechanics force field or knowledge-based algorithm for generating and optimising binding poses, (ii) use of an approximate objective function (usually a docking score) as a surrogate measure of binding affinity, (iii) approximation of a rigid target receptor structure, and (iv) limited synthetic tractability of the designed compounds. We therefore developed the FEgrow software as an open-source, interactive Jupyter notebook based workflow for building user-defined congeneric series of ligands in protein binding pockets to start to address some of these open questions (Fig. 1A).^15^ FEgrow grows user-defined functional groups (R-groups) off a constrained core of a known hit compound, thus incorporating input from structural biology and the expertise of the user in selecting synthetically tractable elaborations. Since publication, we have added functionality for connecting R-groups to the core *via* a flexible linker, which can be chosen from a library of those common to bioactive molecules.^16^ In this way, users can choose from 1 M+ combinations of linker and R-group from our distributed libraries (or upload their own R-group modifications). The modular workflow allows for the incorporation of state-of-the-art molecular modelling algorithms, such as the use of hybrid machine learning/molecular mechanics (ML/MM) potential energy functions to optimise the ligand binding pose,^17,18^ and the gnina convolutional neural network scoring function to predict the binding affinity.^19^ We plan to expand the range of available optimisation algorithms and scoring functions as they become available (see Methods section).

While interactive work is useful for small-scale studies, we have found it useful to automate the workflow for use on high performance computing (HPC) clusters, and since publication have added an application programming interface (API) to FEgrow (Fig. 1B). This enables us to build virtual libraries with a common core, for example, using reaction-based generative scaffold decoration with LibInvent^21^ or substructure searching of compound libraries,^22^ and then rapidly build the compounds into the protein binding pocket with FEgrow. However, unless the libraries are designed using information from the binding pocket, time is wasted building and scoring compounds that are unlikely to be beneficial and it is still not feasible to routinely scan all possibilities.

Hence, rather than exhaustive or random searches of chemical space, we investigate here the use of active learning to elaborate compound design with FEgrow. The general idea behind this approach is that a subset of compounds is evaluated using an expensive design objective function (in this case the molecular growing and scoring algorithms in FEgrow) and used to train a machine learning model (Fig. 1C).^23^ The machine learning model then predicts the objective function for the remainder of the chemical space, and the next subset of molecules is picked for evaluation (for example, in order to optimise the objective or further explore the chemical space). By cycling through this procedure, the algorithm can iteratively make up for any lack of diversity in the initial training subset, and it is has been found previously that the most promising compounds can be identified by evaluating only a fraction of the total chemical space.

Several studies have investigated the effects of choices such as machine learning algorithm, sample selection protocol and total dataset size on active learning efficiency for experimental and computational affinity predictions.^24–28^ In general, active learning has been shown to increase enrichment of hits compared to either random or one-shot training of a machine learning model, at low additional cost, and to be relatively insensitive to choices of molecular representation, model hyperparameters and initial training subsets. Active learning has shown practical utility in prioritising compounds based on objective functions from docking^29–31^ or free energy calculations.^25,26,32,33^

Here, we interface FEgrow with active learning to efficiently search the chemical space of linkers and R-groups from a user-defined vector. As well as using a docking score to guide optimisation, we also experiment with functions that combine other molecular properties, such as molecular weight, and 3D structural information, such as protein–ligand interaction profiles (PLIP).^34^ To address the issue of synthetic tractability of the compound designs, we combine the workflow with regular searches of the Enamine REAL database to ‘seed’ the chemical search space with promising purchasable compounds. After testing and optimising the hyperparameters of the active learning models, we apply the algorithm to the prospective design of inhibitors of the main protease (MPro) of SARS-CoV-2, the virus responsible for the COVID-19 pandemic. This target has undergone extensive study in recent years. The COVID moonshot consortium used open science crowd-sourced designs, in combination with high-throughput structural biology and assays, free energy calculations, and machine learning driven synthetic route predictions, to generate a series of potent inhibitors.^4^ Other notable approaches that include biological confirmation of hits have employed, for example, structure-based design starting from a drug repurposing study,^20^ virtual screening of a curated collection of commercially available compounds,^35^ a deep reinforcement learning model using pharmacophore and substructure matches with known inhibitors,^36^ and a deep generative framework using only target sequence information as input (along with prioritisation based on factors such as docking score and retrosynthetic feasibility).^37^ Here we employ active learning to prioritise compounds for purchase and testing from the Enamine REAL database based only on early fragment hits. We suggest several novel designs that show activity in a fluorescence-based Mpro assay, as well as automatically generating several compounds that show high similarity to known moonshot hits.

## Methods

### Workflow design

The FEgrow software package is described in detail elsewhere.^15^ Briefly, FEgrow aims to grow a ligand within a protein binding pocket, starting from a provided receptor structure, ligand core and growth vector (Fig. 1A). Libraries comprising 2000 linkers^16^ and around 500 R-groups, are provided, or users can supply their own. Merging is achieved using the RDKit package,^38^ which also generates an ensemble of ligand conformations *via* the ETKDG algorithm,^39^ with the atoms of the core strongly restrained to the input structure. That is, the default behaviour is to allow flexibility only in the regions of the grown linkers and R-groups. The ensemble of ligand structures is filtered to remove any that clash with the protein, and the remaining conformers are structurally optimised in the context of a rigid protein binding pocket using the OpenMM software.^18^ During energy minimisation, the protein is treated using the AMBER FF14SB force field,^40^ while intramolecular energetics of the ligand are described, where possible, using the ANI-2x machine learning potential.^17^ Non-bonded interactions between the protein and ligand are described using a mechanical embedding scheme, that is, they use electrostatics and Lennard-Jones terms described by either the Open Force Field ‘Sage’^41^ or GAFF2 (ref. 42) general force fields. The goal of this hybrid machine learning/molecular mechanics approach is to correct for known deficiencies in potential energy surfaces of classical force fields, while ensuring that optimisations are significantly faster than using full QM/MM.

The lowest energy structures are then output for scoring. In the first iteration of FEgrow, we used the gnina convolutional neural network (CNN), which has been jointly trained on binding pose and affinity prediction.^19,43,44^ We showed that the gnina ‘CNNaffinity’ scores (predicted pK) correlated reasonably well with experiment for ten series of congeneric inhibitors built using FEgrow.^15^ Here, we add further options for scoring molecules based on protein–ligand interaction profile (PLIP),^34^ molecular properties, or a combination thereof. For construction of the PLIP score, interactions formed in the available protein-fragment complex crystal structures were one-hot encoded to form a reference vector of desired interactions (here, hydrophobic, hydrogen-bonding, π-stacking, and salt bridge were all identified). A similar vector was constructed for the designed *de novo* compound, and its Tanimoto similarity to the reference vector used as the objective for optimisation. It has been argued that combining information from various properties can also be advantageous,^8^ for example by using pharmacophore constraints in combination with docking scores, and we make use here of a simple, combined score (CS):1
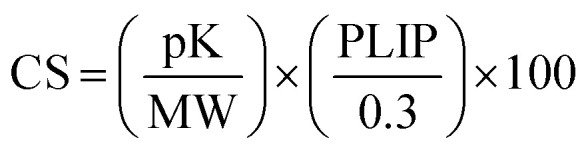
which aims to maximise the predicted gnina affinity (pK) and the protein–ligand interaction profile (PLIP) similarity to reference structures, while keeping the molecular weight (MW) low.

### Active learning

Active learning^23^ is a subset of machine learning that is based on iteratively labeling data points from an unlabeled dataset (in our case, *de novo* compounds that are built into protein binding pockets and scored). The aim is to pick the most useful samples for training a surrogate model, whilst ultimately minimising the potentially expensive computation needed to find instances that maximise an objective function. There are two main components to an active learning workflow: the regression model, and the acquisition function. Every scored instance is used to train a specified machine learning model, with more examples refining the model accuracy, which is then used to select new molecules to be built. In this work we consider and benchmark two models.

The first approach is gradient boosting machine (GBM), which is a random forest based technique, utilising ensembles of decision trees. These trees are created from random subsets of features (fingerprints), that are then used to make predictions. GBMs expand on traditional random forests by using the gradient of the error to construct trees specifically designed to minimise this error. Gradually increasing the number of relatively poor individual trees additively increases their predictive power (hence ‘gradient boosted’). The second model is Gaussian process (GP) regression, which is a Bayesian approach that makes predictions by assuming observations can be modelled by the probability distribution over possible reasonable (Gaussian) functions.^45^ These Gaussian distributions are iteratively refined by the observation of new samples. Because model prediction is performed *via* a probability distribution, it natively incorporates uncertainty and other useful quantities, such as estimates of expected improvement of a given new sample.^46^

The acquisition function defines the method by which new molecules are picked at the start of each active learning cycle, with the simplest example being a ‘greedy’ approach, which directly selects the (currently predicted) highest scoring molecules. However, an acquisition function has to balance picking the best compounds, with the need to further refine the accuracy of the machine learning model. Picking the best scoring candidates in descending order might initially increase the objective function, but the algorithm will have the propensity to get stuck in local maxima and to be sensitive to the initial selection of training molecules.

There are a variety of alternatives that aim to avoid the problems of a simple greedy approach, and the approach used here is the upper confidence bound (UCB) uncertainty-based acquisition function.^47^ UCB considers not just the value of the objective function, but also the variance of the prediction (model uncertainty), effectively biasing towards the selection of molecules about which the model is the least certain of the predicted score. The UCB function is defined by:

2 UCB(*x*) = *μ*(*x*) + *βσ*(*x*),

where *μ*(*x*) and *σ*(*x*) are the mean and standard deviation of the regressor for molecule *x*, and *β* is a parameter controlling the degree of exploration (high *β* increases the chances that a molecule with moderate score but high uncertainty will be picked). The effects of the choice of machine learning model and acquisition function, as well as other active learning hyperparameters, are discussed later.

### Database search

A challenge for automated growing of linkers and R-groups, and for *de novo* design in general, is the synthetic tractability of the designed compounds. Approaches to address this limitation could include a synthetic accessibility score in the objective function^48^ or the expert curation of libraries with known synthetic routes.^32^ However, we wished to fully automate the design process, and be confident of acquiring compounds for rapid design-make-test-analyse cycles. We therefore make use of the rapidly-growing make-on-demand compound libraries as a surrogate measure of synthetic accessibility. Ideally, we might use the entire catalogue as a chemical space in which to perform the active learning. Although such an approach has been used as a one–off screen,^49^ evaluating the regression models used here soon becomes prohibitively expensive in an active learning cycle. On the other hand, highly efficient methods have been developed for similarity and substructure searches of these libraries.^22^ We therefore make use of these searches to seed the chemical space with compounds that are similar to the predicted actives at each step of the active learning cycle (Fig. 1C). In this way, at the subsequent acquisition step, we enable the algorithm to pick compounds for growing and scoring that are likely to be scored highly (due to similarity with other highly scoring compounds) and available for purchase or synthesis (due to presence in on-demand libraries).

In detail, the Enamine REAL database of 4.5 B compounds was searched for similarity to designed molecules through the public interface to SmallWorld https://sw.docking.org, using a graph-edit-distance space search.^22^ At each cycle, 100 new, top-scoring compounds were searched, and up to 100 of the most similar compounds from the REAL database were extracted per search query (using a maximum distance of 5 steps). This 10 K compound set was filtered for substructure match with the core using RDKit,^38^ and those compounds that passed were added to the active learning search space. Active learning then selects compounds for scoring following Enamine enrichment, as usual, but there is no explicit bias to select compounds from the on-demand catalogue.

### Computational details

Protein input structures were taken from the set of noncovalent complexes crystallised early during the COVID-19 pandemic.^3^ In particular, the input PDB: 5R83 was used as the receptor structure for active learning design, and chimera was used to add hydrogen atoms.^50^ The ligand was truncated to include only the pyridyl moiety, as this appeared in other available crystallised fragments in a consistent binding mode (PDB: 5RE4, 5REH, 5R84, 5RF3 (ref. 3)) and with a suitable vector for growth into the binding pocket. The full set of 23 non-covalent complexes (that had ligands bound in areas of the pocket accessible by a growth vector) was additionally used for construction of the reference PLIP^34^ interactions.

For testing of the active learning protocols, the chemical space was assembled by combining the pyridyl moiety with 508 R-groups^51^ and 100 of the most common linkers^16^ from the FEgrow library. A total of 47 710 unique molecules were successfully grown into the binding pocket and scored using the gnina CNN scoring function.^19^ A further 1656 molecules were assigned a penalty score of pK = 0 as they could not be embedded due to steric clash with the protein. In cases where rare errors occurred, such as a failure to assign force field parameters, the molecules were discarded completely.

The previously tested FEgrow molecule building protocol was applied throughout.^15^ The ETKDG algorithm^39^ was used to generate 50 conformers, using a 0.5 Å root-mean-square similarity threshold. Any conformers with an atom closer than 1 Å to any atom in the protein was discarded. Energy minimisation was applied using a hybrid machine learning/molecular mechanics energy function in a mechanical embedding scheme.^15^ The ANI-2x potential^17^ was used for the ligand, in cases where all elements in the molecule are covered by the model, or the Open Force Field Sage^41^ potential otherwise. The lowest energy conformer was retained for scoring.

An active learning library based on scikit^52^ and modAL^53^ python packages was adopted from another study.^26^ A set of molecules to initialise the active learning cycle can be selected *via* RDKit's MaxMin picker^38^ from the chemical space, or picked at random. The processing was parallelised using the python library Dask,^54^ which supports a diverse set of technologies, including the Slurm Workload Manager that is deployed ubiquitously on high-performance computing clusters. Dask is used to secure resources (scheduling workers on Slurm), submitting work and retrieving results. The three major computationally-expensive components were parallelised: (1) building and scoring of the molecules, (2) computing the Morgan fingerprints, and (3) computing the Tanimoto similarity across the chemical space for the Gaussian process modelling.

## Results

### Interfacing FEgrow with active learning enables efficient search of chemical space

**Fig. 2:**
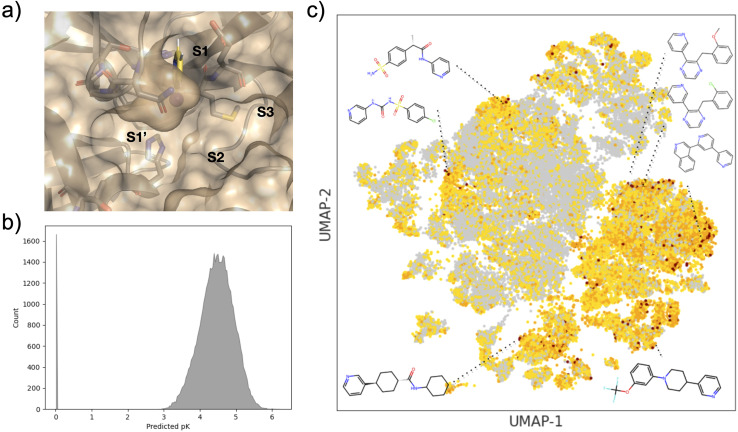
(a) The position of the ligand core and definitions of binding pocket labels, the purple sphere is the hydrogen atom for replacement. (b) Histogram of computed pK for the 47 K compound oracle dataset. (c) UMAP of entire 47 K oracle chemical space, coloured by computed pK. 2D structures of representative strong binders are included.

In order to investigate the performance of the active learning protocol, and the effect of machine learning hyperparameters, we built a labelled ‘oracle’ set of 47 K compounds using standard FEgrow input settings (see Computational details). This is a larger set of compounds than would be typically built and scored against a target, but knowing the affinities of the full chemical space enables us to assess the performance of the active learning approach. The common core was selected to be a pyridyl fragment common to several early crystal structures of the SARS-CoV-2 main protease,^3^ located in the S1 pocket with a vector pointing into the enzyme active site (Fig. 2a).

Fig. 2b shows the distribution of predicted binding affinities, computed using the gnina convolutional neural network scoring function^19^ from FEgrow built structures. The scores are symmetrically distributed around pK = 4.5, with a maximum affinity of around 6.0, which is indicative of a set of low molecular weight (range between 100 and 350 Da, Fig. S1†), unoptimised compounds at the start of a hit finding effort. Indeed, it is at this stage where the options for expansion are vast, and strategies to suggest exploration of hits are particularly valuable. Note that compounds that could not be built (for example, due to steric clashes with the protein) are arbitrarily assigned a pK of zero, so that this information can be included in the active learning model.

Fig. 2c further shows the UMAP projection of the chemical space, coloured by predicted pK. The visualisation shows a diverse composition of linkers and functional groups, with well-spread clusters of the highest affinity binders, potentially providing a challenging search space for active learning. Fig. 2b also shows locations in the chemical space of example linker and R-groups, attached to the pyridyl core, that make up the stronger predicted binders. Favourable predicted linkers include amides, sulfonylurea and various 6-membered ring heterocycles, and relatively bulky R-groups are feasible, which is generally expected given the size and shape of the binding pocket.^3,4^ (Note that at this stage no consideration is given to synthetic accessibility or stability of the compound designs).

We next sought to use active learning to accelerate the search through this chemical space, using the oracle to assess the performance of model hyperparameters, and using the predicted binding affinity as the optimisation target. In particular, we have investigated the effects of initial compound selection (random or diverse), number of compounds picked per cycle (in the range 200–500), machine learning model (GBM or GP) and acquisition method (greedy or UCB). As discussed, the dependence of active learning efficiency on the choice of model parameters is well documented, and so we do not devote much space to it here.

**Fig. 3:**
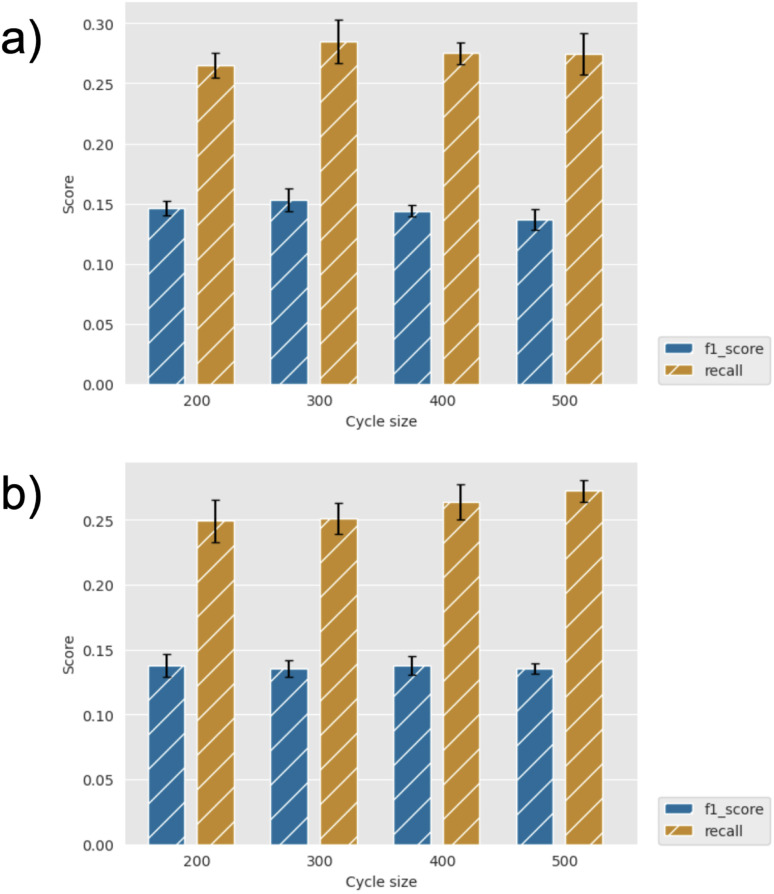
Recall and F1 score for diverse initial selection (a) GBM and (b) GP models, and greedy acquisition for identification of top 2% scoring compounds for different cycle sizes. Error bars show standard errors over five runs.

**Fig. 4:**
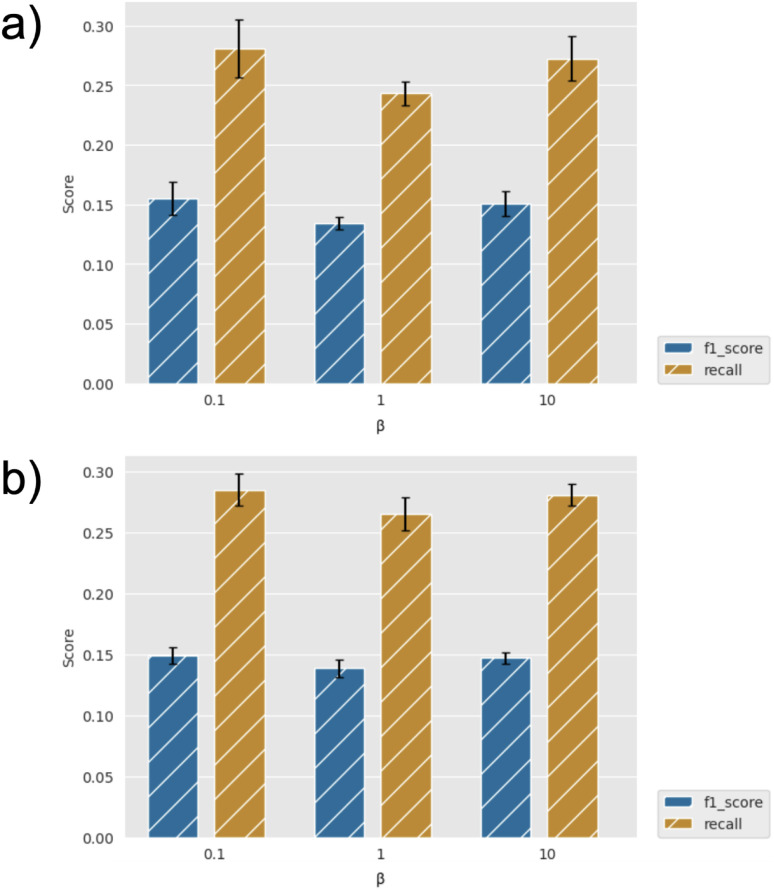
Recall and F1 score for diverse initial selection using GP and UCB acquisition (and varying *β*) with cycle sizes of (a) 200 and (b) 400 for identification of top 2% scoring compounds. Error bars show standard errors over five runs.

By way of example, Fig. 3 shows the effect of the number of compounds picked per cycle on model recall and precision (F1 score) for the two machine learning models (GBM and GP). For a fixed total number of compounds selected (here, 2500), one might expect the model to improve at small sample sizes (hence, more active learning cycles), but we find that the efficiency is already well converged when picking 500 per cycle. Similarly, the choice of machine learning model has little effect, with slightly higher metrics for the GBM model, but both recall and precision comparisons are within the error bars. Fig. 4 further shows the effect of using the UCB uncertainty-based acquisition function, instead of greedy selection, in conjunction with the GP machine learning model. There is some small improvement in recall over greedy selection, but no significant change in the metrics used either as a function of cycle size or the *β* parameter in eqn (2). Fig. S4–S7† show the results of additional hyperparameter experiments, with similar conclusions.

Note that for the current dataset, random selection would give a recall of 0.05 and F1 score of 0.03 for identification of the top 2% of compounds. Therefore, with recall of around 0.25–0.30 for most of our experiments, we see efficiency improvements with active learning of around a factor of 5× compared to random selection. For reference, the growth and scoring of this compound set in FEgrow requires around 1000 cpuhrs, which is not prohibitive, but automated acceleration at no cost is clearly worthwhile.

**Fig. 5:**
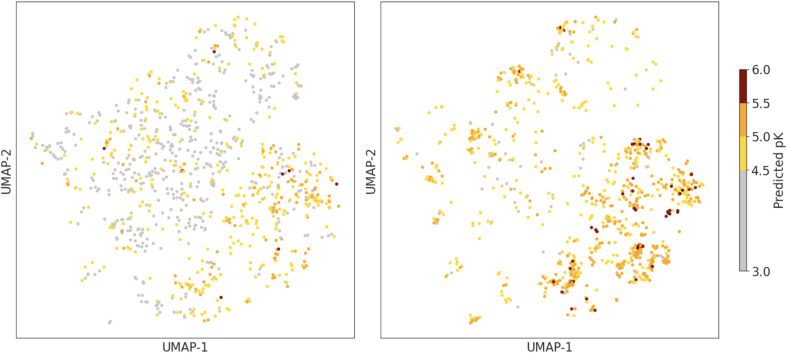
Difference in selection for first (left) and final (right) active learning cycles, showing a narrowing into areas predicted to be potent and avoiding unpromising areas.

In the next section, we choose to use a GP model with UCB acquisition function, with a cycle size of 200 and a diverse set of starting compounds. The overall accuracy of the chosen regression model (using *β* = 10), following training on 5% of the dataset, is 0.97 pK units (Fig. S2†), which is competitive with typical models used in active learning with fingerprint-based representations.^27^Fig. 5 shows a similar UMAP projection as in Fig. 2, but now only showing compounds acquired by our chosen active learning model in the first (left) and final (right) cycles. We observe both a wide exploration of the chemical space, which is important to increase diversity in the final set, and a focusing of the explored regions in the final cycle to compounds with a higher predicted binding affinity, which is important for the use of the model to identify strong binders.

### Active learning driven fragment expansion identifies potential SARS-CoV-2 MPro inhibitors

Having established that the active learning protocols tested here are able to improve the efficiency of chemical space searches with FEgrow, we turn now to prospective design of potential noncovalent SARS-CoV-2 MPro inhibitors. A wealth of computational and experimental data has been generated for this target in recent years, but here we limit ourselves to structural information that was available in the early months of the COVID-19 pandemic. In particular, as in the previous section, we consider expansion of the pyridyl fragment (PDB: 5R83) along a vector into the binding pocket containing the catalytic cysteine (Cys145).^3^ We now expand the size of the chemical space to an initial 250 000 molecules, built from the combination of supplied libraries of 500 linkers and 500 R-groups, such that full building and scoring of the space is prohibitively expensive for routine study. To address the issue of synthetic feasibility of the output designs, we add an additional step in the active learning cycle (Fig. 1C), whereby the chemical space is periodically seeded with compounds from the REAL database that are similar to the highest scoring compounds (see Methods). Fig. S3† demonstrates successful incorporation of the Enamine compounds into the active learning cycles, with a significant fraction of the built and scored compounds originating from this source.

**Fig. 6:**
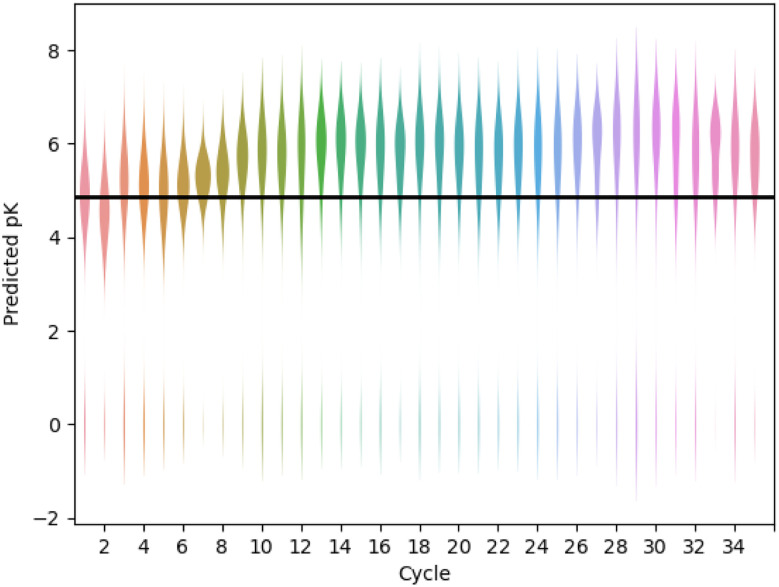
Active learning drives improvements in predicted binding affinity. A GP model is used, with UCB acquisition function (*β* = 0.1), a cycle size of 200 and a diverse set of starting compounds. The solid horizontal line shows the average score for 377 compounds randomly selected from the REAL database that were built with FEgrow.

Fig. 6 shows an example design run, optimising the compounds for predicted pK using the gnina scoring function (further examples are given in Fig. S8–S10†). The distribution of predicted affinity increases over the first 10 active learning cycles then starts to saturate with a mean predicted pK close to 6 (micromolar affinity). Over the full run, 95% of the compounds were successfully built (assigned pK > 0) and 15% had a predicted pK > 6. For comparison, we also extracted 1000 molecules at random that contained the pyridyl substructure from the REAL database used to seed the active learning cycles. For this set, 377 molecules (38%) could be successfully built, with an average predicted pK = 4.9 and only two compounds with predicted pK > 6.0 (0.2%).

**Fig. 7:**
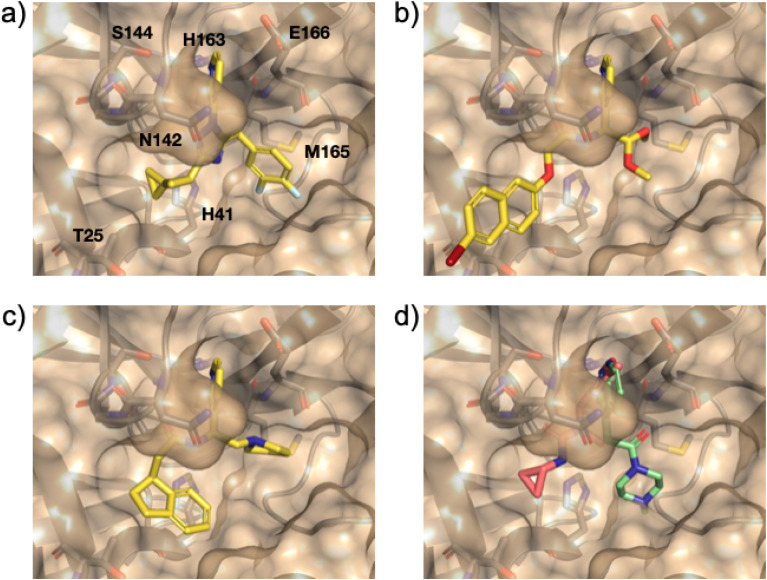
(a) Top-scoring compounds optimised for (a) predicted pK, (b) protein–ligand interaction profile and (c) combined scoring function. (d) Fragment 5RGI shown in pink (H-bond donation by Gly143, Ser144, Cys145 and His163), and 5RF7 in green (hydrophobic and H-bond donation with Glu166).

Fig. 7a shows the highest scoring compound from this run, with a predicted affinity of 88 nM. The compound extends hydrophobic contacts into the S3 and S1′ pockets, for example with Met165 and Thr25, but despite this does not form any specific polar interactions (other than the original core interaction with His163). Since an early fragment screen had provided valuable information about the nature of potential protein–ligand interactions in this binding pocket, we sought to reduce the reliance on the gnina scoring function and drive the active learning towards compounds that recovered known crystallographic information (see Methods). Fig. 7b shows the top-scoring compound, as defined by the Tanimoto similarity to the vector of reference interactions. In this case, the grown molecule forms additional hydrogen bonding interactions with Asn142, Gly143, Ser144, Cys145 and Glu166, and hydrophobic interactions with Thr25 and Glu166. The majority of these interactions are recapitulated by, for example, fragments PDB: 5RGI and 5RF7 (Fig. 7d).

Finally, we sought to combine the strengths of both docking scores and crystallographic information to optimise a combined scoring function. Fig. 7c shows the top-scoring compound as defined by eqn (1) after 33 cycles of active learning. Although this compound is scored much lower by the gnina scoring function (predicted affinity 2 μM), it extends into the S3 and S1′ pockets and retains many of the interactions observed in Fig. 7b (*e.g.*, hydrogen bonding interactions with Asn142, Gly143, Ser144, Cys145 and Glu166).

### Analysis of hit compounds

**Fig. 8:**
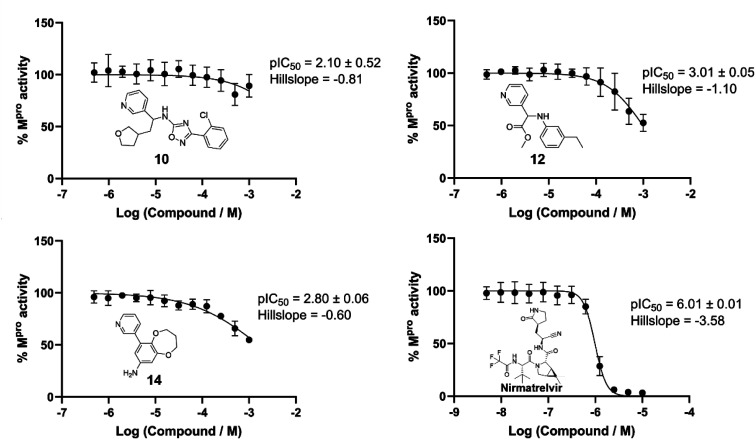
IC_50_ determination of selected compounds with Mpro. Compounds 10, 12 and 14 were tested at a top concentration of 1000 μM. Nirmatrelvir was tested at a top concentration of 10 μM as a positive control. Datapoints presented as mean ± SD; pIC_50_ presented as mean ± SEM; two biological repeats consisting of three technical replicates. 10 consists of one biological repeat with three technical replicates. Conditions: Mpro (0.2 μM), 12 hour pre-incubation with compounds, 20 μM fluorescent substrate, 50 mM Tris-HCl (pH 7.3), 1 mM EDTA and temperature 25 °C.

**Fig. 9:**
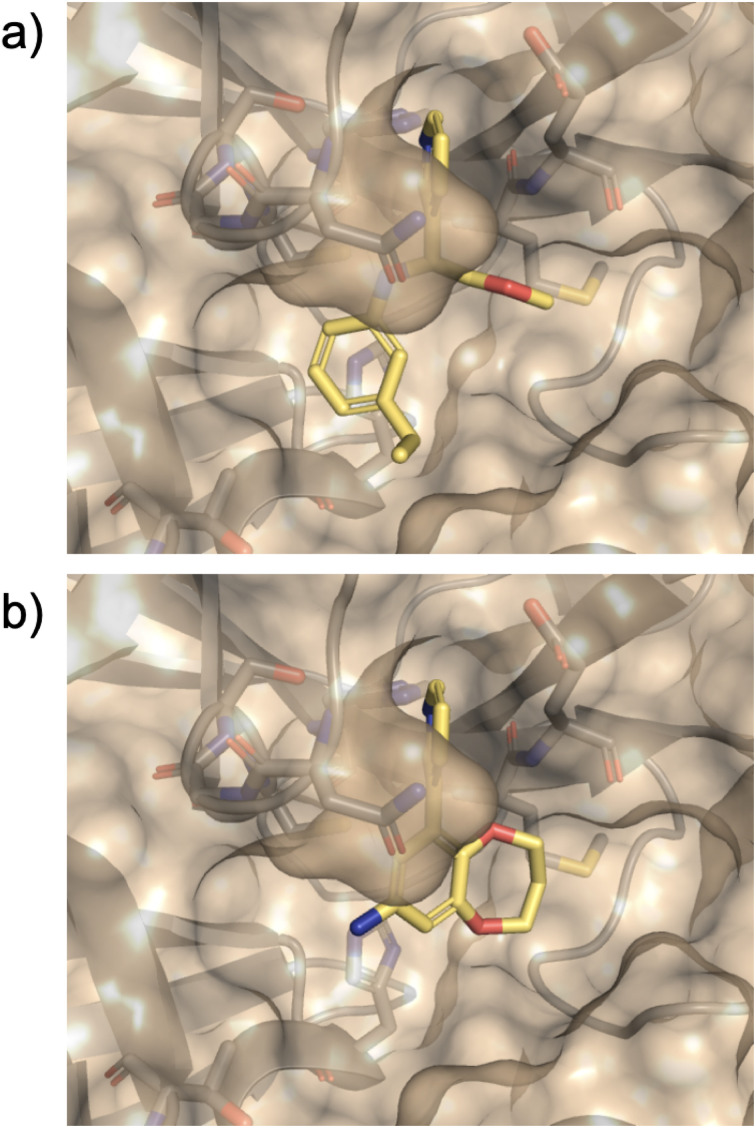
Predicted bound structures of compounds (a) 12 (Z1470573089) and (b) 14 (Z8969017446).

The top 500 compounds from each of four active learning runs (two optimising predicted pK, one optimising protein–ligand interactions, and one optimising the combined scoring function) were checked for availability from the Enamine store. Interestingly, very few of the top scored by predicted pK were available (four in total). This is likely due to an important unavailable building block(s), and could be mitigated in future by increasing diversity and/or including direct store queries in the search process. In any case, we focussed here on outputs from the remaining two runs, and submitted the top 10 protein–ligand interaction and top 25 combination scoring compounds for costing. Finally, a total of 19 designed compounds were purchased (of which 15 had been optimised using the combination score) based on quoted price and excluding similar compounds (based on visual inspection). Two control compounds were also included; one known binder from a crystallographic fragment screen (Enamine ID: Z44592329; PDB: 5R83)^3^ and one elaborated compound from the COVID moonshot study (Enamine ID: Z4943052515 (literature IC_50_ 0.288 μM)).^4^ The twenty one purchased compounds (Fig. S11†) were evaluated in a fluorescence-based Mpro activity assay at 1000, 500, 10 μM (Fig. S12†). Compounds 5 and 6 were excluded from the study due to solubility issues at 1000 μM in the assay conditions. Five compounds (8, 10, 12, 14 and 21 (the positive control^4^)) showed reduction of Mpro activity ≤50% at 1000 μM. The IC_50_ values of these compounds, except 8 which displayed background autofluorescence, were further determined (Fig. 8). Compounds 10, 12 and 14 showed a concentration-dependent inhibition of Mpro activity (measured pIC_50_ 2.10, 3.01, 2.80 respectively). Nirmatrelvir, an orally bioavailable antiviral drug targeting Mpro, showed inhibition (pIC_50_ 6.01), which was slightly higher than the reported IC_50_ (0.022 μM^55^), likely due to the limit of the assay (the enzyme concentration was at 0.2 μM). Fig. 9 shows the predicted structures of compounds 12 and 14 from the active learning design runs. Both compounds form hydrogen bonding interactions with the backbone of Glu166, as well as hydrophobic interactions in the S1′ pocket.

**Fig. 10:**
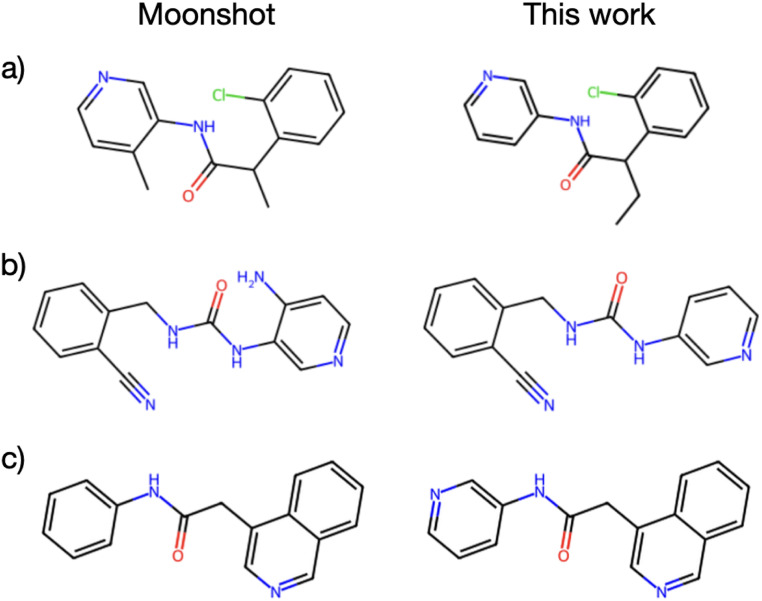
(a) Experimental moonshot compound (literature IC_50_ 17 μM) and most similar compound from this study, from active learning optimisation of predicted pK (*β* = 10), (b) experimental moonshot compound (literature IC_50_ 54 μM) and most similar compound from this study, from active learning optimisation of predicted pK (*β* = 10), (c) experimental moonshot compound (literature IC_50_ 57 μM) and most similar compound from this study, from active learning optimisation of combination scoring function.

Finally, to investigate whether the relatively low affinity of designed compounds is due to insufficient exploration of chemical space or the empirical objective functions used to optimise molecules, we performed a retrospective analysis of the designed compound space against known binders resulting from the COVID moonshot crowd-sourced discovery campaign.^4^ In particular, Fig. 10 shows the three most similar compounds from the active learning runs (as defined by Tanimoto similarity search between RDKit Morgan fingerprints with a radius of 3 and size of 2048) to a curated set of 292 hit compounds. Considering that our FEgrow runs took as input only a single PDB receptor structure and pyridyl fragment core, it is clear that this fragment growing and on-demand library screening approach holds promise for suggesting biologically active compounds early in hit discovery campaigns. However, further work is needed to ensure that the most promising compounds are located at the top of ranked lists for synthetic prioritisation and testing.

## Discussion and conclusions

In this study, we have combined the FEgrow software, an open modular workflow for building and scoring ligands in protein binding pockets, with active learning to guide and automate chemical space searches for promising binders. In agreement with numerous other studies,^27^ we have shown that search efficiency is not too dependent on the hyperparameters of the active learning model, which include the choice of regression model, the acquisition function and number of compounds picked per cycle. For this particular study, we find efficiency improvements of a factor of around 5× over random selection, which will aid throughput of future prospective design efforts. We use as an initial chemical search space a naive enumeration, off a user-defined core, of the linkers and R-groups from supplied libraries. We envisage that this initial search space could be made more focused in future, using the methods that we discussed in the Introduction for *de novo* hit expansion, linking and merging,^9–11^ or protocols for generating libraries of synthetically-accessible compounds.^56,57^ That is, the user could enumerate a library of promising compounds, and rapidly build and score the most promising binders using the structure-based design workflows in FEgrow.

With the design of FEgrow, we hope to overcome some of the current limitations of *de novo* drug design discussed in the Introduction. Some of these limitations are addressed in the current study, and some will be addressed in future aided by ongoing advances in molecular modelling and machine learning. For example, we tackle the question of binding pose optimisation by using a fast and accurate machine learning potential (ANI-2x^17^) to describe the ligand energetics in a hybrid ML/MM mechanical embedding scheme. However, with the flexibility of the FEgrow interface with OpenMM,^18^ new models could be substituted in, and these are now approaching sufficient speed and accuracy (including for long-ranged interactions) such that geometry optimisation of the entire protein–ligand complex could be implemented using a single, consistent machine learning potential.^58,59^ In this study, we made the approximation that the protein binding pocket is rigid and used a single receptor structure for design. However, now that ligand building and scoring is fully automated, future studies could use, for example, ensembles of receptor structures, which may be beneficial in cases where the pocket is more flexible.

A limitation of this and other similar studies is the choice of objective function in the active learning cycles. To demonstrate the flexibility of the FEgrow package, we demonstrated four design cycles here, two optimising for predicted affinity using the gnina CNN scoring function and two including a more direct optimisation of protein–ligand contacts extracted from crystallographic fragment screens. While we do not have enough data to assess the relative merits of these scoring functions, we expect the latter to be useful where experimental structural data exists, at least as part of a multi-objective optimisation in future.^60^ As a flexible alternative to PLIP scores trained on system-dependent crystal structures, it has also been shown that transferable neural networks can be trained on the PDBbind structural database to recognise favourable protein–ligand interactions.^61^

As shown in Fig. 1c, to address the issue of synthetic tractability of the *de novo* built compounds, we inserted regular queries of the Enamine REAL database into the active learning cycles. In this way, we can use the initial chemical space to train the active learning regression models, and then over time seed the chemical space with compounds that are both similar to predicted actives and purchasable. In this way, we were able to test the predictions of the active learning workflow with a turn around time of a few weeks from order to biological testing. We note that the methods described here are not intended to be specific to the REAL database, and expanding the search query to a range of suppliers would further increase the similarity between top-scoring designs and seeded compounds. Furthermore, the question of how to efficiently search these very large databases is undergoing continual scrutiny, and methods that employ 3D similarity searches^62,63^ or that formulate the search in synthon-based rather than enumerated form^64,65^ could be incorporated into FEgrow in future. Of the 19 designed compounds that were purchased here, three showed measurable activity, but none approached the desired levels for further progression. Nevertheless, a similarity search showed the presence of effective inhibitors in the built chemical space, and so further investigation will focus on ranking compound designs ahead of purchase. Such post-processing could include molecular dynamics simulations to assess stability of predicted binding modes with a flexible receptor, or ideally an extra stage of physics-based free energy calculations to more rigorously predict binding affinities of compound designs.^26,66^

While the test case employed here is intended to showcase possible applications of FEgrow in prospective computer-aided design, it is challenging to comprehensively benchmark the utility of new computational methods. In this regard, the CACHE (critical assessment of computational hit-finding experiments) provide an invaluable resource for prospectively validating computational predictions.^67^ Indeed, we entered an earlier version of FEgrow into CACHE challenge 2, with some success in identifying a chemical starting point for SARS-CoV-2 NSP13.^68^ To facilitate further design and validation work by the community, we have made FEgrow freely available, along with a full tutorial for the active learning workflow, at: https://github.com/cole-group/FEgrow.

## Data availability

FEgrow is freely available, with a set of tutorials, at https://github.com/cole-group/FEgrow. ESI† data including oracle and prospective active learning datasets and scripts used to perform ancillary data analysis are available at https://github.com/cole-group/FEgrow_AL_data.

## Author contributions

Ben Cree: conceptualisation, data curation, formal analysis, investigation, methodology, software, validation, visualisation, writing – original draft. Mateusz Bieniek: conceptualisation, data curation, formal analysis, investigation, methodology, software, validation, visualisation, writing – original draft. Siddique Amin: data curation, formal analysis, investigation, methodology, writing – original draft. Akane Kawamura: resources, supervision, writing – review & editing. Daniel Cole: conceptualisation, funding acquisition, methodology, project administration, resources, supervision, writing – original draft, writing – review & editing.

## Conflicts of interest

The authors declare no competing interests.

## Supplementary Material

DD-004-D4DD00343H-s001
