# Supplementary material for: Active learning driven prioritisation of compounds from on-demand libraries targeting the SARS-CoV-2 main protease
Source: Digit Discov. 2025 Jan 8;4(2):438–50. doi: 10.1039/d4dd00343h (PMC11726688; doi:10.1039/d4dd00343h)
Supplement: DD-004-D4DD00343H-s001 [file DD-004-D4DD00343H-s001.pdf]

Supplementary Information (SI) for Digital Discovery.
This journal is © The Royal Society of Chemistry 2025

Supplementary Information for: Active learning driven prioritisation of compounds from on-demand libraries targeting the SARS-CoV-2 main protease

Ben Cree,[a] Mateusz K. Bieniek, Siddique Amin, Akane Kawamura, and Daniel J. Cole*

School of Natural and Environmental Sciences, Newcastle University, Newcastle upon Tyne NE1 7RU, United Kingdom

E-mail: daniel.cole@ncl.ac.uk

[a]B.C. and M.K.B. contributed equally to this work.

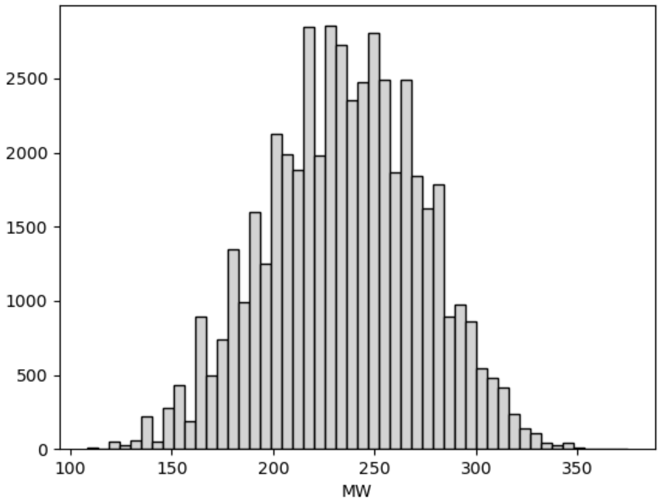

Figure S1: Distribution of molecular weights (MW, Da) for the 47 K compound oracle dataset.

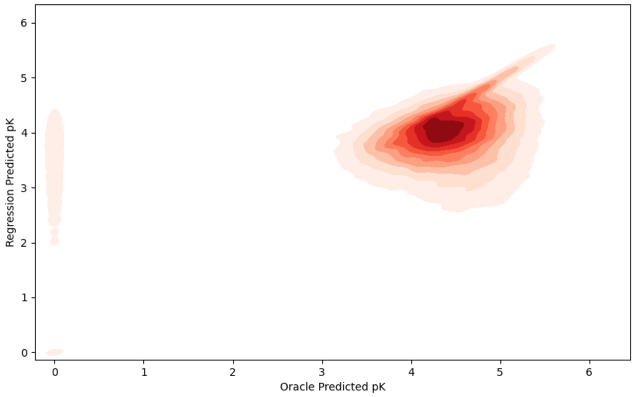

Figure S2: Correlation between the predicted pK using a Gaussian process regression model and the oracle predictions. Overall RMSE between the predictions is 0.97 pK units. (Cycle size = 200, diverse initial selection of molecules, UCB acquisition function, $\beta = 10$).

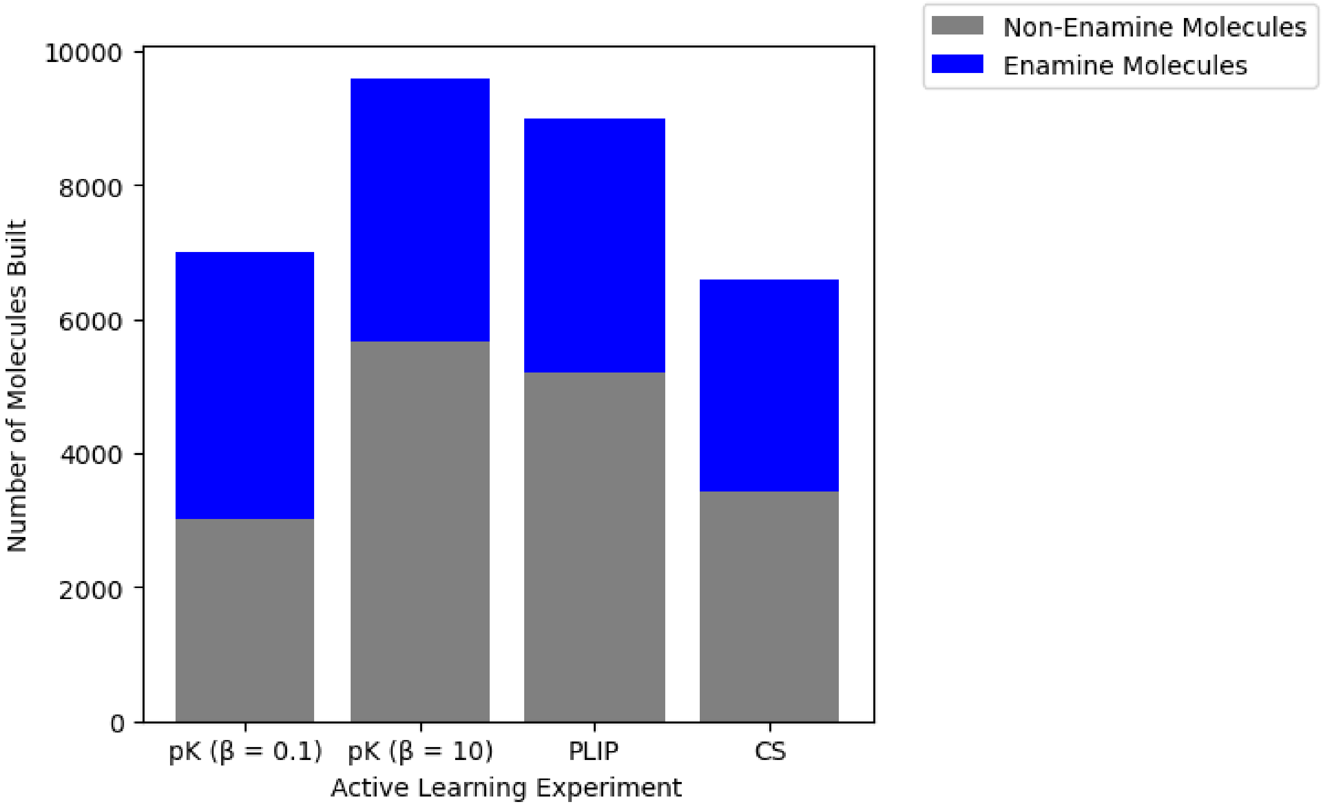

Figure S3: Histogram of the number of Enamine molecules added for each experiment, as a fraction of the total number of molecules built.

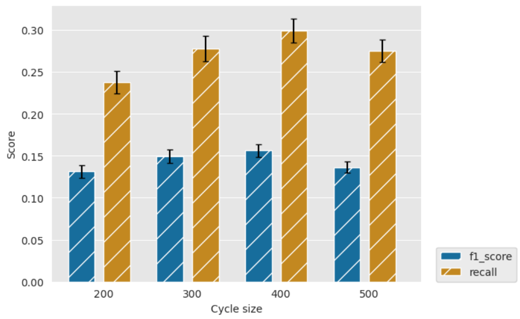

(a) Top 2% activity

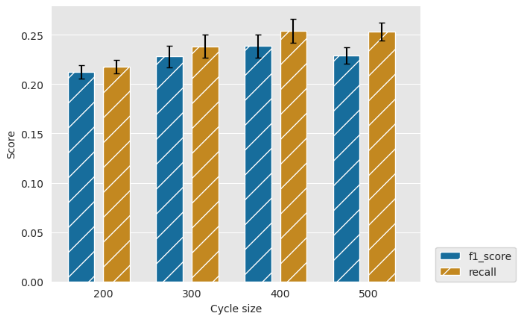

(b) Top 5% activity

Figure S4: F1/recall for Experiment: Random initial molecule selection, GBM regression model and greedy acquisition at 2 and 5 % as a function of different cycle sizes.

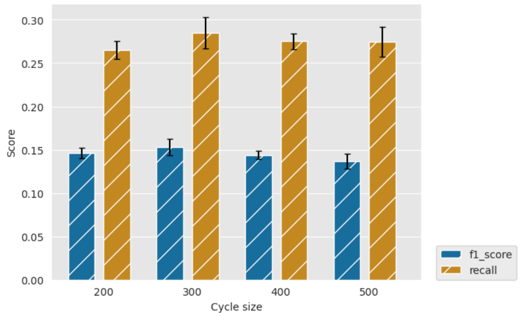

(a) Top 2% activity

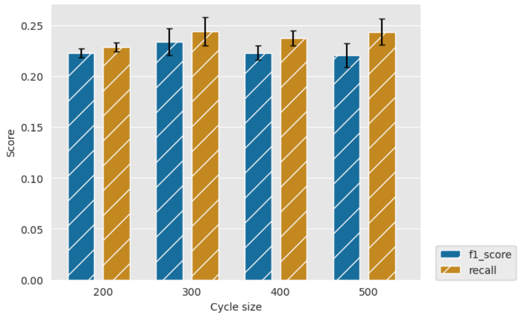

(b) Top 5% activity

Figure S5: F1/recall for Experiment: Diverse (MaxMin) initial molecule selection, GBM regression model and greedy acquisition at 2 and 5 % as a function of different cycle sizes.

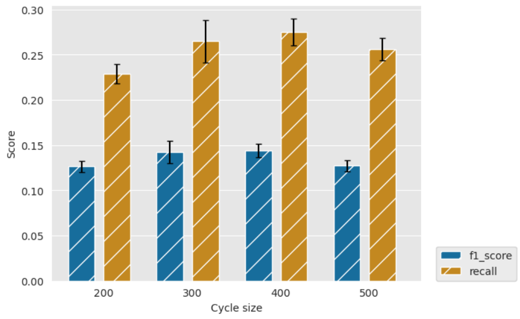

(a) Top 2% activity

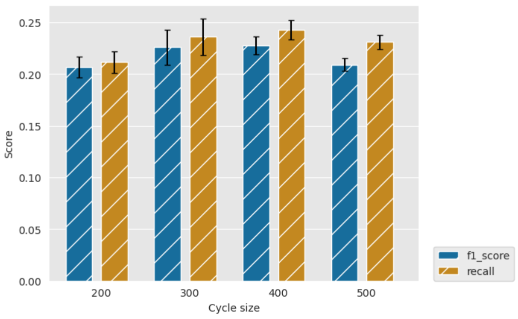

(b) Top 5% activity

Figure S6: F1/recall for Experiment: Random initial molecule selection, GP regression model and greedy acquisition at 2 and 5 % as a function of different cycle sizes.

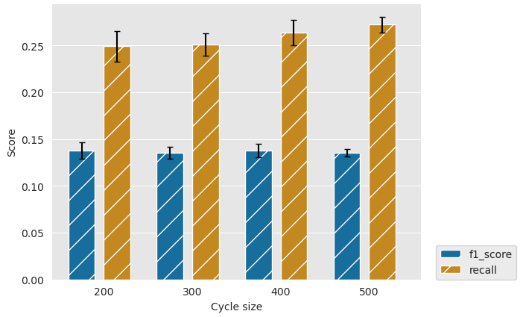

(a) Top 2%

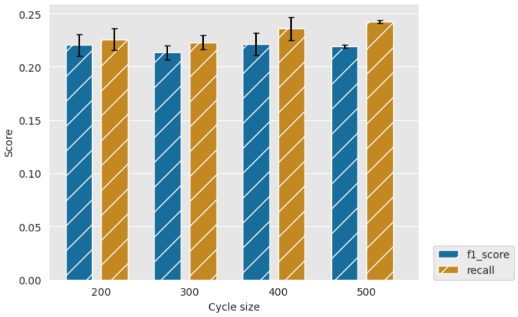

(b) Top 5%

Figure S7: F1/recall for Experiment: Diverse (MaxMin) initial molecule selection, GP regression model and greedy acquisition at 2 and 5 % as a function of different cycle sizes.

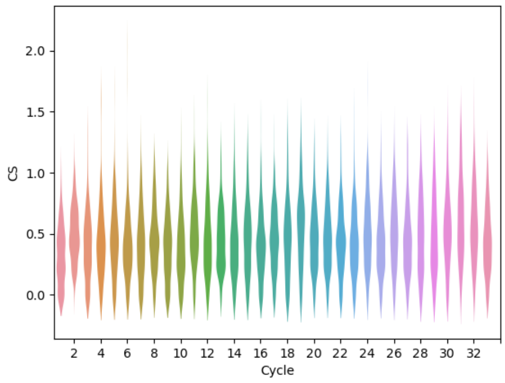

Figure S8: Active learning drives improvements in predicted CS scoring function. A GP model is used, with UCB acquisition function ($\beta = 0.1$), a cycle size of 200 and a diverse set of starting compounds.

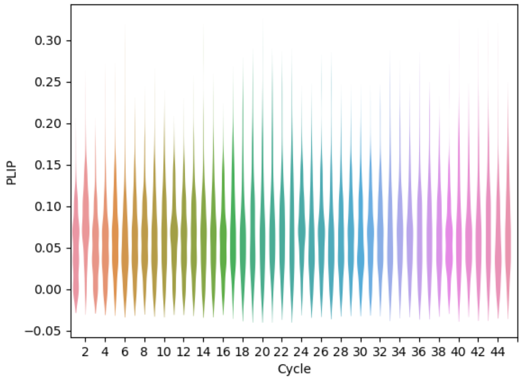

Figure S9: Active learning drives improvements in predicted PLIP scoring function. A GP model is used, with UCB acquisition function ($\beta = 0.1$), a cycle size of 200 and a diverse set of starting compounds.

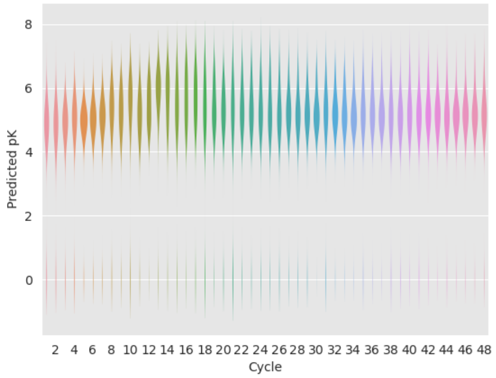

Figure S10: Active learning drives improvements in predicted binding affinity scoring function. A GP model is used, with UCB acquisition function ($\beta = 10$), a cycle size of 200 and a diverse set of starting compounds.

Supplementary Note 1

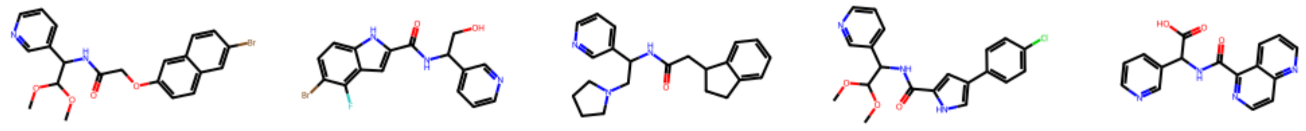

1 - Z1695837227 | 2 - Z8969125232 | 3 - Z5121743676 | 4 - Z1906945801 | 5 - Z8969017233

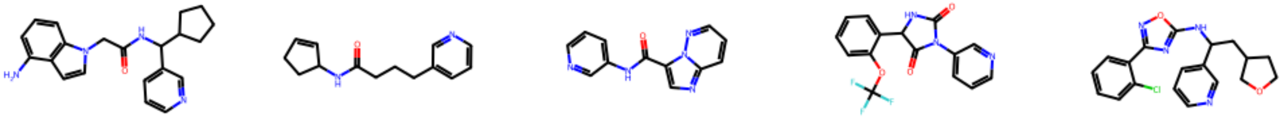

6 - Z5545298658 | 7 - Z2644125118 | 8 - Z5553958262 | 9 - Z8899278291 | 10 - Z5023297106

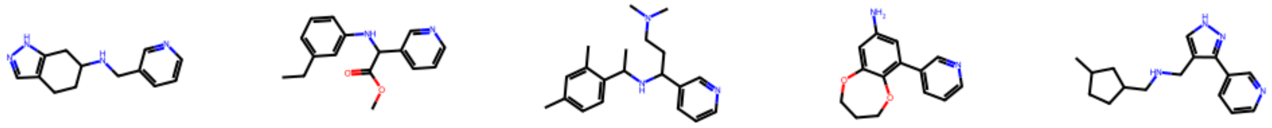

11 - Z1411290446 | 12 - Z1470573089 | 13 - Z2898903751 | 14 - Z8969017446 | 15 - Z1939245981

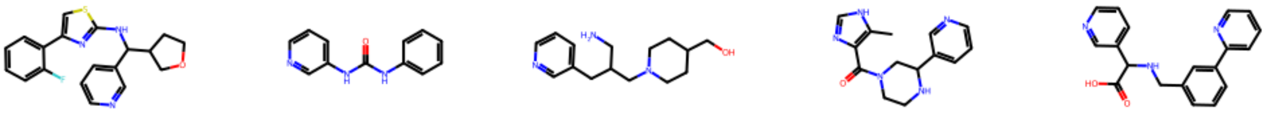

16 - Z5279116450 | 17 - Z44592329 | 18 - Z4591995195 | 19 - Z2801272830 | 20 - Z8969017289

Figure S11: 2D structures of the Enamine compounds ordered, along with their compound number and Enamine IDs. Note that compound **17** is a control compound taken from a previous study.[1]

Experimental

Protein Production and Purification

Recombinant His-tagged Mpro was produced as described[2] in E. coli BL21 (DE3) containing pGEX-6P-1 Mpro plasmid. A 50 mL starter culture was inoculated and grown with

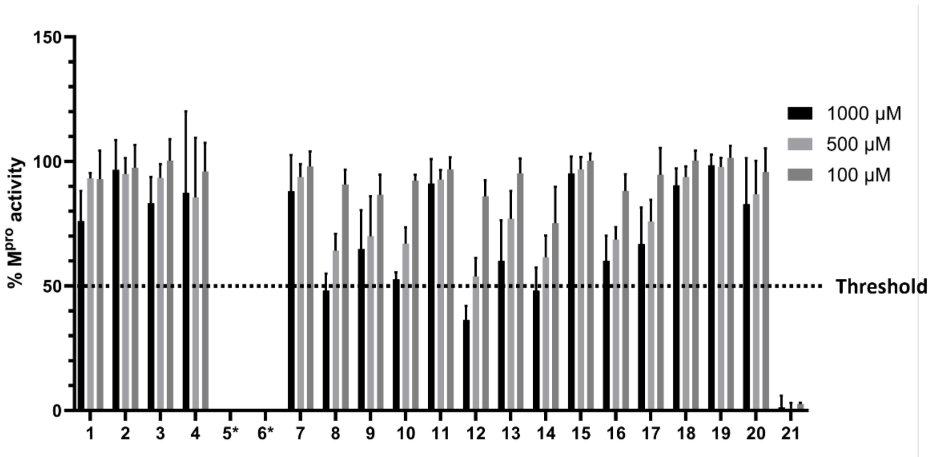

Figure S12: Initial compound screening for inhibition of Mpro enzyme activity. Compounds were tested for inhibition of Mpro catalytic activity at concentrations of 1000 μM, 500 μM and 100 μM. Compounds **17** and **21** were included as controls. Compounds **12** and **14** reduced the Mpro activity below the threshold ($\leq$ 50 % Mpro activity) at 1000 μM and were selected for subsequent IC50 analysis. **8** was not chosen for further analysis due to background auto-fluorescent activity. Data represented as mean $\pm$ SD; 2 biological repeats consisting of 3 technical replicates. 10 consists of 1 biological repeat with 3 technical replicates. Conditions: Mpro (0.2 μM) 12-hour pre-incubation with compounds, 20 μM fluorescent substrate, 50 mM Tris-HCl (pH 7.3), 1 mM EDTA and temp: 25°C. Compounds **5** and **6** were excluded from the analysis due to poor solubility in assay conditions.

carbenicillin (100 μg/mL) in LB broth for 8 hours at at 37 °C with shaking (200 RPM). The expression media (1 L Formedium LB autoinduction media + 10 mL glycerol in a 2.5 L baffled Erlenmeyer flask) was inoculated with 10 mL of the starter culture and grown at 37 °C with carbenicillin (100 μg/mL) at 200 RPM until reaching an OD600 of 0.6. Cells were further incubated for 16 h at 18°C, harvested by centrifugation (8000 g, 10 min) and pellets were frozen at -80 °C. The frozen pellet (12 g) was re-suspended in lysis buffer [50 mM Tris, 300 mM NaCl, pH 8] before being lysed by sonication for 6 mins (5s on, 20s off cycle, 40 % AMP, Sonics VCX-500) twice before subsequent centrifugation (50,000 g, 30 min). The cell lysate was filtered in a 0.45 μm syringe filter and added to a 3 mL bed volume Nickel Sepharose gravity flow column pre-equilibrated with lysis buffer. The column was washed with 72 mL wash buffer [50 mM Tris, 300 mM NaCl, 25 mM imidazole, pH 8] and eluted with 12 mL elution buffer [50 mM Tris, 300 mM NaCl, 500 mM imidazole, pH 8] and collected as 3 mL fractions. Protein-containing fractions were combined and concentrated using a 10 kDa molecular weight cut off concentrator (Amicon Ultra), and subsequently purified by size-exclusion chromatography using a gel filtration column (HiLoad 16/600 Superdex 75pg) on AKTA Pure system in SEC buffer [50 mM Tris, 300 mM NaCl, pH 8]. His-tagged Mpro-containing fractions (>90 % purity by 10 % SDS PAGE gel) were concentrated (478.7 μM), aliquoted and stored at -80 °C.

Fluorescent Activity Assay

Mpro fluorescent substrate peptide (MCA-AVLQSGFR-Lys(Dnp)-Lys-NH2) was purchased from GL Biochem. All assays were performed as described[3] in black 384-well microplates (Greiner Bio-One). Concentrations reported as used in the final assay volume of 30 μL. Compound dilutions were prepared in assay buffer [50 mM Tris-HCl, 1mM EDTA, pH 7.3] with 3 % DMSO (1 % DMSO final). 10 μL compound was incubated with 10 μL Mpro (0.2 μM final) for 30 min at 25 °C before addition of 10 μL of the fluorescent substrate peptide (20 μM final). Fluorescence (330 nm excitation / 390 nm emission) after 20.5 min (at linear

range) at 25 °C (BMG Pherastar FSX) was used to calculate the IC_{50} values. Background fluorescence of compounds **4**, **7**, **8**, **11**, **16** and **21** was subtracted from the raw datapoints. Datapoints were normalised to DMSO control (maximum Mpro activity) and no enzyme control and presented as '% Mpro activity'. Analysis was performed on GraphPad Prism V10 fitted with the model 'log(inhibitor) vs. normalized response, variable slope'. All assays were performed twice in technical triplicates unless stated otherwise.

Supplementary References

(1) Boby, M. L. et al. Open science discovery of potent noncovalent SARS-CoV-2 main protease inhibitors. *Science* **2023**, *382*, eabo7201.

(2) Douangamath, A. et al. Crystallographic and electrophilic fragment screening of the SARS-CoV-2 main protease. *Nature Communications* **2020**, *11*, 5047.

(3) Jin, Z. et al. Structure of Mpro from SARS-CoV-2 and discovery of its inhibitors. *Nature* **2020**, *582*, 289–293.
